# Phenotypic and Genotypic Antimicrobial Resistance in Non-O157 Shiga Toxin-Producing *Escherichia coli* Isolated From Cattle and Swine in Chile

**DOI:** 10.3389/fvets.2020.00367

**Published:** 2020-07-10

**Authors:** Nicolás Galarce, Fernando Sánchez, Verónica Fuenzalida, Romina Ramos, Beatriz Escobar, Lisette Lapierre, Esteban Paredes-Osses, Gabriel Arriagada, Raúl Alegría-Morán, Nilton Lincopán, Danny Fuentes-Castillo, Alejandra Vera-Leiva, Gerardo González-Rocha, Helia Bello-Toledo, Consuelo Borie

**Affiliations:** ^1^Departamento de Medicina Preventiva Animal, Facultad de Ciencias Veterinarias y Pecuarias, Universidad de Chile, Santiago, Chile; ^2^Departamento de Salud Ambiental, Instituto de Salud Pública de Chile, Santiago, Chile; ^3^Instituto de Ciencias Agroalimentarias, Animales y Ambientales, Universidad de O'Higgins, San Fernando, Chile; ^4^Facultad de Ciencias Agropecuarias, Universidad Pedro de Valdivia, Santiago, Chile; ^5^Departamento de Microbiología, Instituto de Ciências Biomedicas, Universidade de São Paulo, São Paulo, Brazil; ^6^Departamento de Patologia, Faculdade de Medicina Veterinária e Zootecnia, Universidade de São Paulo, São Paulo, Brazil; ^7^Laboratorio de Investigación en Agentes Antibacterianos, Facultad de Ciencias Biológicas, Universidad de Concepción, Concepción, Chile; ^8^Millenium Nucleus on Interdisciplinary Approach to Antimicrobial Resistance, Santiago, Chile

**Keywords:** antimicrobial resistance, Shiga toxin, *Escherichia coli*, drug resistance, cattle, swine

## Abstract

Non-O157 Shiga toxin-producing *Escherichia coli* (STEC) is a zoonotic pathogen that causes bloody diarrhea and hemolytic-uremic syndrome in humans, and a major cause of foodborne disease. Despite antibiotic treatment of STEC infections in humans is not recommended, the presence of antimicrobial-resistant bacteria in animals and food constitutes a risk to public health, as the pool of genes from which pathogenic bacteria can acquire antibiotic resistance has increased. Additionally, in Chile there is no information on the antimicrobial resistance of this pathogen in livestock. Thus, the aim of this study was to characterize the phenotypic and genotypic antimicrobial resistance of STEC strains isolated from cattle and swine in the Metropolitan region, Chile, to contribute relevant data to antimicrobial resistance surveillance programs at national and international level. We assessed the minimal inhibitory concentration of 18 antimicrobials, and the distribution of 12 antimicrobial resistance genes and class 1 and 2 integrons in 54 STEC strains. All strains were phenotypically resistant to at least one antimicrobial drug, with a 100% of resistance to cefalexin, followed by colistin (81.5%), chloramphenicol (14.8%), ampicillin and enrofloxacin (5.6% each), doxycycline (3.7%), and cefovecin (1.9%). Most detected antibiotic resistance genes were *dfr*A1 and *tet*A (100%), followed by *tet*B (94.4%), *bla*_TEM−1_ (90.7%), *aac(6)-Ib* (88.9%), *bla*_AmpC_ (81.5%), *cat*1 (61.1%), and *aac(3)-IIa* (11.1%). Integrons were detected only in strains of swine origin. Therefore, this study provides further evidence that non-O157 STEC strains present in livestock in the Metropolitan region of Chile exhibit phenotypic and genotypic resistance against antimicrobials that are critical for human and veterinary medicine, representing a major threat for public health. Additionally, these strains could have a competitive advantage in the presence of antimicrobial selective pressure, leading to an increase in food contamination. This study highlights the need for coordinated local and global actions regarding the use of antimicrobials in animal food production.

## Introduction

Shiga toxin-producing *Escherichia coli* (STEC) is a zoonotic pathotype of *E. coli* recognized as an important cause of food-borne illness worldwide. Several animal species are reservoirs of STEC strains, mainly cattle with a reported prevalence of up to 70.1% in beef cattle (1) and up to 68.7% in swine (2). STEC can cause severe gastroenteritis, hemorrhagic colitis, and life-threatening hemolytic-uremic syndrome (HUS) in children (3, 4), and extrarenal manifestations in adults and the elderly, such as thrombotic thrombocytopenic purpura (5). Among these different illnesses caused by STEC infection, HUS is the most severe, as it has a 2% mortality rate during the acute phase (5), and is considered the main cause of acute renal failure in children, with about 30% of them developing chronic kidney disease (6).

Global incidence of STEC infections in people was estimated in a previous study, which showed that this pathogen is responsible for 2,801,000 acute infections annually, with 3,890 HUS cases and 230 deaths (7). In this context, and according to official data, the incidence of HUS in Chile is 3.2/100,000 in children under 4 years, with a mortality rate of 3–5% (8, 9).

The O157 serogroup is the most frequently associated with outbreaks and sporadic cases of HUS in people (10, 11), although other serogroups such as O26, O45, O103, O111, O121, and O145, have also been associated with severe disease (11, 12). In addition, the economic costs associated with STEC infections also have a high impact. In this context, it has been estimated that average economic losses in the United States reach US$ 896/case and US$ 101 million for non-O157:H7 STEC infections, and that combined economic losses for public health and food agriculture are estimated at US$ 993 million per year (13).

Antibiotic treatment of STEC infections in humans is not recommended, as there is evidence that treatment may worsen the disease by inducing toxin-related tissue damage and symptoms in patients (14). However, toxin production depends on the type and concentration of the drug used (15). During the O104:H4 outbreak in Germany, patients treated with azithromycin at the acute phase showed decreased STEC carriage periods (16), while no patients treated with azithromycin for long-term STEC shedding developed HUS (17). Although antibiotic therapy is not recommended for STEC infections, multidrug-resistant (MDR) strains constitute a public health concern, both for human and veterinary medicine, as these strains contribute to the resistance gene reservoir that can be easily exchanged among different bacterial species either in the host or in the environment (18).

It is widely accepted that extensive use of antimicrobials in animal production systems is a major driver of multi-drug resistance in bacteria (19). Furthermore, long-term subtherapeutic exposure to antibiotics can result in mutation enrichment and/or acquisition of mobile genetic elements such as plasmids, transposons, and integrons that can confer a phenotype of increased resistance to these compounds (20). The presence of antibiotic-resistant bacteria in animals and food, regardless of their pathogenicity, constitutes a public health risk as the genetic pool from which bacterial pathogens can acquire antibiotic resistance has increased in the environment (21).

STEC strains resistant to β-lactams, aminoglycosides, phenicols, and tetracyclines, among others, have been isolated from livestock worldwide, together with their resistance-encoding genes and integrons (22, 23). These studies indicate variable antimicrobial resistance (AMR) levels in the STEC isolates according to geographic area, possibly due to control policies in the use of these compounds in animal husbandry. However, international trade of animals and their products can enable the transmission of strains and/or their resistance genetic determinants among countries. In addition, new resistance patterns have emerged in *E. coli* strains, being colistin resistance one of the most important threats to public health worldwide (24).

As part of a larger study, cattle and swine were screened for STEC as previously published (25), recovering culturable STEC strains at a frequency of 17% in cattle and 1% in swine. The aim of this study was to characterize the phenotypic and genotypic AMR of the isolated strains, to assess the potential impact in public health and contribute updated data to national and international AMR surveillance programs.

## Materials and Methods

### Bacterial Strains

During 2018, samples from intestinal content of cattle and swine (*n* = 300, each) at four abattoirs located in the Región Metropolitana were obtained. From these samples, 54 STEC strains were isolated from cattle (*n* = 51) and swine (*n* = 3) (25). Strains were stored in trypticase soy broth (Oxoid, Basingstoke, UK) mixed with glycerol (1:1, v/v) at −80°C. Sampling, processing, bacterial identification and characterization were detailed in a previous study (25).

### Phenotypic Antimicrobial Resistance

AMR of all isolated strains was quantified by a minimal inhibitory concentration (MIC) test using the VITEK2 system (bioMérieux, Marcy-l'Étoile, France) and the AST-GN98 card according to the manufacturer's instructions, and clinical cut-off values were applied according to the Clinical and Laboratory Standards Institute guidelines (26). The cards included aminoglycosides (amikacin and gentamicin), β-lactams (amoxicillin-clavulanic acid, ampicillin, cefalexin, cefovecin, cefpodoxime, ceftazidime, ceftiofur, and imipenem), folate synthesis inhibitors (trimethoprim-sulfamethoxazole), nitrofurans (nitrofurantoin), phenicols (chloramphenicol), quinolones (ciprofloxacin, enrofloxacin, and marbofloxacin), tetracyclines (doxycycline), and also cefepime, cefotaxime, ceftazidime alone, and in combination with clavulanic acid for the detection of extended-spectrum β-lactamase (ESBL). Colistin resistance was determined with the broth microdilution method (27–29), analyzing eight antibiotic concentrations (32-0.25 μg/mL). *E. coli* ATCC 25922 was used as quality control and *E. coli* NCTC 13846 as positive control. MDR was confirmed if an isolated strain presented resistance to three or more antibiotics of different classes (30). Intermediate strains were classified as resistant.

### Genotypic Antimicrobial Resistance

The presence of 12 AMR genes in all STEC strains was assessed by PCR in a LifeECO® Thermocycler (Hangzhou Allsheng Instruments Co, Hangzhou, China). For DNA extraction, an inoculum of each strain plated on MacConkey agar plates (Oxoid, Basingstoke, UK) and incubated at 37°C for 18–24 h was resuspended in sterile plastic tubes containing 500 μl of sterile nuclease-free water and boiled for 15 min at 100°C. Subsequently, tubes were centrifuged at 26,480 g for 5 min at room temperature. In parallel, plasmid DNA was obtained using the E.Z.N.A.® Plasmid DNA Mini Kit II (Omega Bio-Tek, Norcross, GA, USA), following manufacturer's instructions. Concentration and quality of the obtained DNA was measured in a NANO-400 micro-spectrophotometer (Hangzhou Allsheng Instruments Co). Samples with a 260/280 nm absorbance ratio close to the optimal range (1.8–2.0) were kept at −20°C for further analyses (31). The genes analyzed included *bla*_TEM−1_, *bla*_CTX−M_, chromosomal *bla*_AmpC_ and *bla*_NDM1_ for β-lactams; *aac(6)-Ib* and *aac(3)-IIa* for aminoglycosides; *tet*A and *tet*B for tetracyclines; *cml*A and *cat*1 for phenicols; and *dfr*A1 for folate synthesis inhibitors (32–39). To detect the presence of colistin resistance genes, eight types of *mcr* genes were analyzed (*mcr*1-*mcr*8) following previous protocols (40, 41). Additionally, class 1 and class 2 integrons were detected by conventional PCR (42). All PCR reactions were performed in duplicate. Gene selection was based on their distribution in *E. coli* and their clinical impact in both animal and public health, under the concept of One Health (24, 43, 44). Strains belonging to our collection, whose PCR products for the detection of the aforementioned genes were sequenced and their nucleotide identity corroborated by comparison to sequences deposited at GenBank® (National Center for Biotechnology Information, Bethesda, MD, USA) (data not published), were used as positive controls. Table 1 summarizes all primers used for molecular detection of AMR genes.

**Table 1:** Oligonucleotide sequences for antimicrobial resistance genes and integrons, expected product size, and references.

**Gene**	**Primers (5^**′**^-3^**′**^)**	**Expected product size (bp)**	**References**
*bla*_TEM−1_	F: ATCAGCAATAAACCAGC R: CCCCGAAGAACGTTTTC	516	(33)
*bla*_CTX−M_	F: ATGTGCAGYACCAGTAARGTKATGGC R: TGGGTRAARTARGTSACCAGAAYCAGCGG	593	(36)
*bla*${}_{\text{AmpC}}^{*}$	F: TTCTATCAAMACTGGCARCC R: CCYGTTTTATGTACCCAYGA	500	(35)
*bla*_NDM1_	F: GGTTTGGCGATCTGGTTTTC R: CGGAATGGCTCATCACGATC	621	(39)
*aac(6)-Ib*	F: TTGCGATGCTCTATGAGTGGCTA R: CTCGAATGCCTGGCGTGTTT	482	(37)
*aac(3)-IIa*	F: CGGAAGGCAATAACGGAG R: TCGAACAGGTAGCACTGAG	740	(34)
*tet*A	F: GGTTCACTCGAACGACGTCA R: CTGTCCGACAAGTTGCATGA	577	(32)
*tet*B	F: CCTCAGCTTCTCAACGCGTG R: GCACCTTGCTGATGACTCTT	634	(32)
*cml*A	F: CCGCCACGGTGTTGTTGTTATC R: CACCTTGCCTGCCCATCATTAG	698	(38)
*cat*1	F: AGTTGCTCAATGTACCTATAACC R: TTGTAATTCATTAAGCATTCTGCC	547	(34)
*dfr*A1	F: AAGAATGGAGTTATCGGGAATG R: GGGTAAAAACTGGCCTAAAATTG	391	(34)
*mcr*1	F: AGTCCGTTTGTTCTTGTGGC R: AGATCCTTGGTCTCGGCTTG	320	(40)
*mcr*2	F: CAAGTGTGTTGGTCGCAGTT R: TCTAGCCCGACAAGCATACC	715	(40)
*mcr*3	F: AAATAAAAATTGTTCCGCTTATG R: AATGGAGATCCCCGTTTTT	929	(40)
*mcr*4	F: TCACTTTCATCACTGCGTTG R: TTGGTCCATGACTACCAATG	1,116	(40)
*mcr*5	F: ATGCGGTTGTCTGCATTTATC R: TCATTGTGGTTGTCCTTTTCTG	1,644	(40)
*mcr*6	F: GTCCGGTCAATCCCTATCTGT R: ATCACGGGATTGACATAGCTAC	566	(41)
*mcr*7	F: TGCTCAAGCCCTTCTTTTCGT R: TTCATCTGCGCCACCTCGT	892	(41)
*mcr*8	F: AACCGCCAGAGCACAGAATT R: TTCCCCCAGCGATTCTCCAT	667	(41)
*intI1*	F: GGGTCAAGGATCTGGATTTCG R: ACATGGGTGTAAATCATCGTC	483	(42)
*intI2*	F: CACGGATATGCGACAAAAAGGT R: GTAGCAAACGAGTGACGAAATG	788	(42)

* *Chromosomally encoded bla_AmpC_*.

### Statistical Analysis

For the phenotypic AMR characterization, multiple correspondence analysis (MCA) was used to evaluate the proximal relationships of the resistant/susceptible conditions among the different antibiotics tested. MCA is a non-parametric technique for assessing the pattern of relationships among several categorical variables by identifying a reduced number of orthogonal dimensions that capture most variability present in the original variables (45). The same statistical analyses were performed to assess the relationship of the presence or absence of AMR genes among the isolates. In all cases, MCA analyses were limited to the derivation of two dimensions as a preliminary analysis indicated that these captured a substantial amount of the total variance, and were performed only with variables that presented variability (i.e., antibiotics, genes). The relationships among the antibiotics' resistant/sensitive condition, and among the presence/absence condition of resistance genes were graphically assessed by the construction of two-dimensional correspondence maps. All MCA-related analyses were performed using IMB© SPSS© Statistics v.26 (IBM Corp., Armonk, NY).

## Results

### Phenotypic Antimicrobial Resistance Characterization

All 54 strains analyzed were phenotypically resistant to at least one antibiotic, all being resistant to cefalexin (100%, *n* = 54), followed by colistin (81.5%, *n* = 44), chloramphenicol (14.8%, *n* = 8), ampicillin and enrofloxacin (5.6%, *n* = 3), doxycycline (3.7%, *n* = 2), and cefovecin (1.9%, *n* = 1). A 14.8% of the strains were MDR. No ESBL production was detected in any strain, nor resistance to amoxicillin-clavulanic acid, cefpodoxime, ceftazidime, ceftiofur, imipenem, amikacin, gentamicin, ciprofloxacin, marbofloxacin, nitrofurantoin, or trimethoprim-sulfamethoxazole. Table 2 shows the MIC_50_ and MIC_90_ of the STEC strains for the analyzed antibiotics. All strains isolated from cattle were resistant to cefalexin (100%, *n* = 51), followed by colistin (80.4%, *n* = 41), chloramphenicol (11.8%, *n* = 6), ampicillin (3.9%, *n* = 2), and cefovecin (2%, *n* = 1). Additionally, five strains (9.8%) were MDR. On the other hand, all strains isolated from swine were resistant to cefalexin, enrofloxacin, and colistin (*n* = 3), followed by doxycycline and chloramphenicol (*n* = 2), and ampicillin (*n* = 1). All strains of swine origin were MDR (*n* = 3). Table 3 shows the different phenotypic resistance profiles in the STEC strains analyzed, being the cefalexin-colistin resistant phenotype the most frequently detected (66.7%, *n* = 36).

**Table 2:** MICs of selected antimicrobials against STEC strains isolated from cattle and swine.

**Antimicrobial class**	**Antimicrobial**	**MIC_**50**_ (μg/mL)**	**MIC_**90**_ (μg/mL)**	**Range (μg/mL)**
Aminoglycosides	AMK	≤2	≤2	≤2
	GEN	≤1	≤1	≤1
β-lactams	AMC	≤2	≤4	≤2–8
	AMP	≤4	≤8	≤2–≥32
	LEX	8	≤16	8–16
	CFO	≤0.5	≤1	≤0.5–≥8
	CPD	≤0.25	≤0.5	≤0.25–1
	CAZ	≤0.12	≤0.25	≤0.12–0.25
	CFT	≤1	≤1	≤1
	IPM	≤0.25	≤0.25	≤0.25
Folate synthesis inhibitors	SXT	≤20	≤20	≤20
Nitrofurans	NIT	≤16	≤16	≤16
Phenicols	CHL	≤8	≤16	4-≥64
Polymyxins	CST	≤4	≤8	1–16
Quinolones	CIP	≤0.06	≤0.06	≤0.06
	ENR	≤0.12	≤0.12	≤0.12–1
	MRB	≤0.5	≤0.5	≤0.5–1
Tetracyclines	DOX	≤1	≤1	≤0.5–≥16

*MIC_50_ and MIC_90_ are those concentrations required to inhibit growth of 50 and 90% of the isolates, respectively. Amikacin, AMK; amoxicillin-clavulanic acid, AMC; ampicillin, AMP; cefalexin, LEX; cefovecin, CFO; cefpodoxime, CPD; ceftazidime, CAZ; ceftiofur, CFT; chloramphenicol, CHL; ciprofloxacin, CIP; colistin, CST; doxycycline, DOX; enrofloxacin, ENR; gentamicin, GEN; imipenem, IPM; marbofloxacin, MRB; nitrofurantoin, NIT; trimethoprim-sulfamethoxazole, SXT*.

**Table 3:** Phenotypic resistance profiles detected in STEC strains isolated from cattle and swine.

**Resistance profile**	**Origin**	**Number of strains (%)**	**Strain ID**
LEX	Cattle	7 (12.9%)	1, 2, 3, 4, 5, 42, 48
LEX/CST	Cattle	36 (66.7%)	6, 7, 8, 12, 14, 15, 16, 17, 22, 23, 24, 25, 26, 27, 28, 29, 30, 31, 32, 33, 34, 35, 37, 38, 44, 45, 46, 47, 49, 55, 57, 58, 60, 61, 63, 64
LEX/CHL/CST	Cattle	3 (5.6%)	9, 36, 40
LEX/CHL	Cattle	3 (5.6%)	13, 19, 39
LEX/AMP/CFO/CST	Cattle	1 (1.9%)	18
LEX/AMP/CST	Cattle	1 (1.9%)	20
LEX/ENR/CST	Swine	1 (1.9%)	67
LEX/AMP/ENR/DOX/CHL/CST	Swine	1 (1.9%)	68
LEX/ENR/DOX/CHL/CST	Swine	1 (1.9%)	69

*LEX, cefalexin; CST, colistin; CHL, chloramphenicol; AMP, ampicillin; CFO, cefovecin; ENR, enrofloxacin; DOX, doxycycline*.

### Genotypic Antimicrobial Resistance Characterization

As for the AMR genes, the most detected were *dfr*A1 and *tet*A (100%, *n* = 54), followed by *tet*B (94.4%, *n* = 51), *bla*_TEM−1_ (90.7%, *n* = 49), *aac(6)-Ib* (88.9%, *n* = 48), *bla*_AmpC_ (81.5%, *n* = 44), *cat*1 (61.1%, *n* = 33), and *aac(3)-IIa* (11.1%, *n* = 6). No strains harboring *bla*_CTX−M_, *bla*_NDM1_, *cml*A, and *mcr*1-8 genes were detected. Both classes of integrons were detected in 5.5% (*n* = 3) of the strains. Among the strains isolated from cattle, the most frequently detected genes were *dfr*A1 and *tet*A (100%, *n* = 51), followed by *tet*B (94.1%, *n* = 48), *aac(6)-Ib* (92.2%, *n* = 47), *bla*_TEM−1_ (90.2%, *n* = 46), *bla*_AmpC_ (80.4%, *n* = 41), *cat*1 (58.8%, *n* = 30), and *aac(3)-IIa* (11.8%, *n* = 6). On the other hand, all strains isolated from swine harbored *cat*1, *dfr*A1, *bla*_TEM−1_, *tet*A, *tet*B, *bla*_AmpC_, class 1, and class 2 integrons (*n* = 3), followed by *aac(6)-Ib* (*n* = 1). Table 4 shows all the genotypic resistance profiles detected according to origin, being the *dfr*A1/*aac(6)-Ib*/*bla*_TEM−1_/*tet*A/*tet*B/*cat*1/*bla*_AmpC_ profile the most frequently detected (33.3%, *n* = 18).

**Table 4:** Genotypic resistance profiles detected in STEC strains isolated from cattle and swine.

**Resistance profile**	**Origin**	**Number of strains (%)**	**Strain ID**
*dfr*A1/*aac(6)-Ib*/*bla*_TEM−1_/*tet*A/*tet*B	Cattle	5 (9.3%)	1, 2, 4, 14, 15
*dfr*A1/*aac(6)-Ib*/*bla*_TEM−1_/*tet*A/*tet*B/*cat*1	Cattle	1 (1.9%)	3
*dfr*A1/*aac(6)-Ib*/*bla*_TEM−1_/*tet*A/*tet*B/*cat*1/*bla*_AmpC_	Cattle	18 (33.3%)	5, 6, 7, 12, 17, 18, 19, 22, 23, 26, 28, 33, 44, 46, 49, 55, 61, 63
*dfr*A1/*aac(6)-Ib*/*bla*_TEM−1_/*tet*A/*tet*B/*cat*1/*bla*_AmpC_/*aac(3)-IIa*	Cattle	5 (9.3%)	8, 13, 24 25, 27
*dfr*A1/*aac*(6)-Ib/*tet*A/*tet*B	Cattle	1 (1.9%)	9
*dfr*A1/*aac(6)-Ib*/*bla*_TEM−1_/*tet*A/*tet*B/*bla*_AmpC_	Cattle	12 (22.2%)	16, 20, 29, 30, 31, 32, 35, 36, 37, 38, 39, 40
*dfr*A1/*aac(6)-Ib*/*bla*_TEM−1_/*tet*A	Cattle	1 (1.9%)	34
*dfr*A1/*aac(6)-Ib*/*bla*_TEM−1_/*tet*A/*tet*B/*bla*_AmpC_/*aac(3)-IIa*	Cattle	1 (1.9%)	42
*dfr*A1/*aac*(6)-Ib/*tet*A	Cattle	1 (1.9%)	45
*dfr*A1/*bla*_TEM−1_/*tet*A/*tet*B/*bla*_AmpC_/*cat*1	Cattle	2 (3.7%)	47, 64
*dfr*A1/*aac(6)-Ib*/*tet*A/*tet*B/*bla*_AmpC_/*cat*1	Cattle	1 (1.9%)	48
*dfr*A1/*tet*A/*tet*B/*bla*_AmpC_/*cat*1	Cattle	1 (1.9%)	57
*dfr*A1/*tet*A/*tet*B/*cat*1	Cattle	1 (1.9%)	58
*dfr*A1/*aac(6)-Ib*/*bla*_TEM−1_/*tet*A/*cat*1/*bla*_AmpC_	Cattle	1 (1.9%)	60
*dfr*A1/*bla*_TEM−1_/*tet*A/*tet*B/*cat*1/*intI1*/*intI2*	Swine	2 (3.7%)	67, 69
*dfr*A1/aac(6)-Ib/*bla*_TEM−1_/*tet*A/*tet*B/*cat*1/*intI1*/*intI2*	Swine	1 (1.9%)	68

### Statistical Analysis

**Figure 1:**
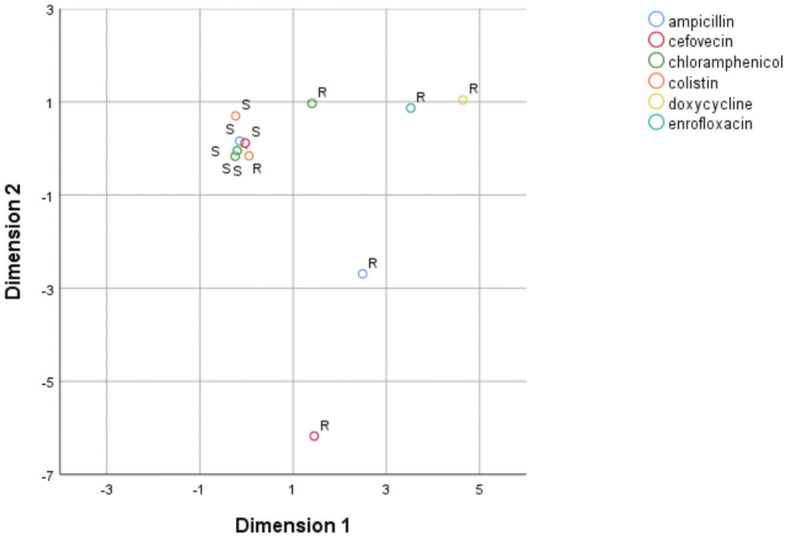
Two-dimension correspondence map for phenotypic AMR characterization (S, sensitive; R, resistant).

**Figure 2:**
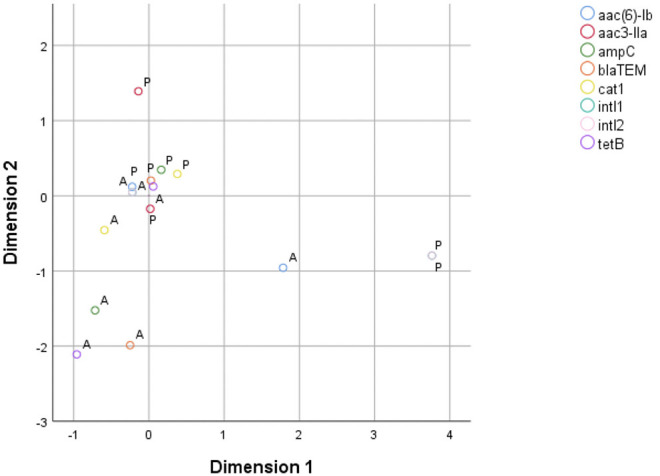
Two-dimension correspondence map for resistance genes (A, absence; P, presence).

MCA for phenotypic AMR characterization included only ampicillin, cefovecin, enrofloxacin, doxycycline, chloramphenicol, and colistin, as there were both resistant and sensitive isolates for each of these antibiotics. The two derived dimensions accounted for 63.73% of total variable variance (first dimension = 38.66%; second dimension = 25.07%). The correspondence map indicated that the first dimension was dominated by isolates resistant to chloramphenicol, enrofloxacin and doxycycline, mostly due to swine isolates; while the second dimension was mainly explained by isolates resistant to cefovecin and, in a lesser extent, to ampicillin (Figure 1). In parallel, MCA to assess the relationship pattern for the presence/absence condition among genes included *bla*_TEM−1_, *bla*_AmpC_, *aac(3)-IIa, aac(6)-Ib, tet*B, *cat*1, *intI1*, and *intI2* genes. The resulting model indicated that the two dimensions accounted for 52.78% of the total variance of the original variables (first dimension = 30.83%; second dimension = 21.95%). Dimension 1 was largely dominated by the presence of *intI1* and *intI2* genes, which belong to swine isolates. Dimension 2 was mostly driven by the presence of *aac(3)-IIa* gene, but also by the presence of *aac(6)-Ib, bla*_TEM−1_, *bla*_AmpC_ and *cat*1 genes. These five genes belonged to bovine isolates (Figure 2).

## Discussion

Gram-negative pathogens like STEC represent a major challenge in Latin America, where MDR, fluoroquinolone-resistant and ESBL-producing strains have spread (46). STEC strains resistant to β-lactams, aminoglycosides, phenicols, and tetracyclines, have been isolated from livestock and humans worldwide (23, 47). However, studies focusing on the AMR of STEC strains isolated from animals in Latin America are scarce. In this context, Ferreira et al. (48) determined the antimicrobial susceptibility of 90 STEC strains isolated from sheep in Brazil, registering 25.5% of resistance to streptomycin, 22.2% to amoxicillin-clavulanic acid, and 19% to nalidixic acid. Furthermore, 6.7% of the strains showed MDR, mainly to gentamicin, streptomycin and tetracycline. The same year, Krüger et al. (49) analyzed the antimicrobial susceptibility of 29 STEC strains of various origins, including 21 strains isolated from cattle in Argentina. Of these 21 strains, only two exhibited resistance against at least one of the drugs analyzed, registering a 9.5% of resistance to ampicillin, amoxicillin-clavulanic acid, cephalothin, and tetracycline, and 4.8% to trimethoprim-sulfamethoxazole, chloramphenicol, and florfenicol. Furthermore, the authors reported the presence of the *bla*_TEM_ gene in the two resistant strains, and of *tet*B, *str*A, *aad*A1, *tet*A, *dfr*A1, *sul*1, *sul*2, *flor*R genes in only one of them. More recently, Amézquita-López et al. (47) evaluated the antimicrobial susceptibility of 59 STEC strains isolated from various domestic animals, including cattle and sheep, in Mexico. Of these strains, 78.0% exhibited resistance to cephalothin, 50.8% to chloramphenicol, 37.3% to kanamycin, 25.4% to ampicillin, 6.8% to amikacin and tetracycline, 3.4% to amoxicillin-clavulanic acid, and 1.7% to cefoperazone, gentamicin, and imipenem. In the other hand, and as far as we know, the present research describes for the first time the phenotypic and genotypic AMR of STEC strains isolated from livestock in Chile, including the characterization of colistin resistance and integron presence.

Antibiotics are usually not prescribed for the treatment of human STEC infections. However, monitoring AMR patterns of intestinal STEC from animal reservoirs, provides valuable information regarding the transmission of resistant strains to humans and of their genetic AMR determinants to other enteric pathogens (22). While most studies have focused on the O157 serogroup (23) AMR in non-O157 STEC strains has increased compared to the former serogroup. In this context, Buvens et al. reported a higher AMR in non-O157 STEC strains than in O157 strains, for ampicillin (23.5 vs. 5.2%), nalidixic acid (10.7 vs. 0%), streptomycin (58 vs. 26%), kanamycin (20 vs. 5%), tetracycline (44 vs. 15%), sulphonamides (59 vs. 22%), and trimethoprim (24 vs. 4%) (50). More recently, a cattle study in Spain reported higher AMR and MDR levels in STEC strains of serogroups O111, O104, O91, and O26 than in serogroup O157 (51). Additionally, AMR acquisition could confer competitive advantages, allowing non-O157 STEC strains to preferentially colonize livestock over other bacterial enteropathogens when there is a selective antimicrobial pressure (18).

Regarding phenotypic AMR in the STEC strains analyzed, our results show that resistance against β-lactams was the most frequent, including cefalexin (100%), followed by polymyxins with an 80.4% of resistance against colistin; phenicols with an 11.8% against chloramphenicol; fluoroquinolones with an 5.6% against enrofloxacin; and tetracyclines with an 3.7% against doxycycline. In this context, Colello et al. reported an 86% of resistance to tetracycline, streptomycin, and chloramphenicol, 71% to trimethoprim/sulfamethoxazole, sulfisoxazole, and ampicillin, and 57% to nalidixic acid in STEC strains isolated from cattle, swine, food and farm environment in Argentina (22), showing higher levels of AMR than those registered here.

Furthermore, in our study the MCA for phenotypic AMR characterization suggests that isolates resistant to doxycycline also present resistance to enrofloxacin, and in a lesser extent to chloramphenicol. If resistance to these antibiotics is present, it is unlikely that the isolates are also resistant to cefovecin or ampicillin. However, these results may be due to the high resistance exhibited by all the three strains of swine origin, so they must be interpreted with caution. In addition, when an isolate is colistin resistant, it is probably sensitive to most of the other antibiotics tested in this study.

Although in Chile there are no official AMR monitoring plans in *E. coli* strains isolated from animals, some studies describe the antimicrobial susceptibility of these isolates in cattle and pigs. In this context, San Martín et al. (52) described the AMR of 50 *E. coli* strains isolated from dairy cattle and 72 strains isolated from beef cattle. Here, strains isolated from the former presented the highest levels of AMR, with 84% of resistance to oxytetracycline, 54% to enrofloxacin, ciprofloxacin and ceftiofur, and a 56% of MDR, being oxytetracycline/enrofloxacin/ciprofloxacin/ceftiofur the most frequently detected phenotypic resistance profile (46%). In contrast, in strains isolated from beef cattle, the highest resistance was to sulfamethoxazole/trimethoprim (10%), followed by oxytetracycline (4%) and ceftiofur (3%), with an 1.4% of MDR, where the most frequent resistance profile corresponded to sulfamethoxazole/trimethoprim (4%). In a more recent study, Hervé-Claude et al. (53) evaluated AMR in 88 *E. coli* strains isolated from calves, where 87.5% were resistant to at least one antimicrobial, 16% showed MDR, and the most frequent resistance profile corresponded to oxytetracycline/sulfamethoxazole/trimethoprim (9.1%). On the other hand, Lapierre et al. (54) evaluated the AMR of 87 strains of *E. coli* isolated from swine, registering 77% of resistance to tetracycline, 74% to streptomycin, and 38% to sulfamethoxazole/trimethoprim (38%), with a 74.7% of MDR, being tetracycline/streptomycin the most frequent resistance profile (33.3%). Good practices in antimicrobial use in Chile, as well as a correct implementation of current policies for antimicrobial use in livestock, could explain the low levels of AMR detected here, compared to previous studies.

β-lactam resistance in STEC strains is well-documented internationally. In this context, Kennedy et al. (18) reported a 53% of resistance to ampicillin, 31% to cephalothin, 16% to ceftiofur, and 6% to cefpodoxime, in non-O157 STEC strains isolated from cattle at farms and abattoirs in Ireland, and an 82% of those strains were MDR. In Latin America, a 100% and a 50% of resistance to ampicillin in non-O157 STEC strains isolated from cattle and swine was reported in Argentina, respectively (22). β-lactams are used in human and veterinary medicine, and are considered of critical importance (3rd and 4th generation cephalosporins, carbapenems, antipseudomonal penicillins, and aminopenicillins with or without β-lactamase inhibitors) and of highly importance (1st and 2nd generation cephalosporins, amidinopenicillins, anti-staphylococcal, and narrow spectrum penicillins) in human medicine, and of critical importance (3rd and 4th generation cephalosporins, penicillins), and of highly importance (1st and 2nd generation cephalosporins) in veterinary medicine (55, 56). Several genes provide resistance against β-lactams by encoding β-lactamases, including *bla*_TEM_, *bla*_NDM1_, and *bla*_AmpC_, among others (57). In this study, only two of these genes were detected in STEC strains isolated from cattle, *bla*_TEM−1_ (90.7%) and *bla*_AmpC_ (81.5%). Similar to our results, Colello et al. detected the *bla*_TEM−1_ gene in 80% of STEC strains isolated from cattle and swine in Argentina, and also the *bla*_AmpC_ gene in an 81.5%, which encodes for a type C β-lactamases (22). Nevertheless, our results are higher than those reported by Kennedy et al., where 43 and 13% of the strains isolated at abattoirs and farms, respectively, harbored the *bla*_AmpC_ gene (18). The high rate of chromosomal *bla*_AmpC_ detected here was expected, as most of *E. coli* strains harbor this gene (58). Although in *E. coli* its expression is constitutive at a low level, overproduction of AmpC due to mutations in the promoter/attenuator leads to resistance to cephalosporins, penicillins, β-lactam-β-lactamase inhibitor combinations and/or aztreonam (58). Furthermore, AmpC production in combination with porin defects may also lead to carbapenem-resistance (59). According to the phenotypic antimicrobial susceptibility registered here, we could infer that these strains are not de-repressed mutants, and maintain their AmpC production at negligible levels. On the other hand, the high rate of *bla*_TEM−1_ detection could explain the resistance of all strains to cefalexin. Conversely, and despite the high rate of detection, only three STEC strains were resistant to ampicillin. This discordant phenotype could be explained by the presence of deficiencies in outer membrane porins, such as OmpC and OmpF. In this context, Choi and Lee (60) analyzed how porins of *E. coli* affect the resistance to several antibiotics, including β-lactams. Thus, they registered an increase in β-lactams resistance in *omp*F mutants, while *omp*C mutants showed variable changes in the MIC to these compounds. More specifically, *omp*F mutants exhibited a 2-fold increase in the MIC of ampicillin, but an 8-fold in the MIC of cefoxitin, while *omp*A and *omp*C mutants did not alter the MIC of the former. Furthermore, triple mutants of the *omp*A, *omp*C, and *omp*F genes showed an 8-fold increase in the MIC of cefoxitin, 4-fold in the MIC of cefalotin, but a decrease in the MIC of ampicillin. These authors pointed out that transport of β-lactams by OmpC and OmpF is the most important factor in bacterial susceptibility to most of these antibiotics, and that this transport could be more important in bacterial susceptibility to ampicillin than to other β-lactams. In the case of the five isolates that did not harbor the *bla*_TEM−1_ gene, their phenotypic resistance against cefalexin could be explained by the presence of other non-ESBL encoding genes, such as *bla*_TEM−2_ (57). In this study, we detected only one strain (strain 18) resistant to cefalexin, cefovecin, and ampicillin, but sensitive to amoxicillin-clavulanic acid and negative for ESBL, which harbored both *bla*_TEM−1_ and *bla*_AmpC_ genes. The amoxicillin-clavulanic acid MIC of this strain was 8 μg/mL, a value that corresponds to the upper limit to be considered sensitive (26). This phenotype could be explained by a low production of AmpC that could confer resistance to at least one expanded-spectrum cephalosporin, but the MIC may not be high enough to classify the strain as resistant (61). However, further studies are needed to elucidate the role of AmpC in this discordant phenotype, using combinations of antibiotic substrates (such as cloxacillin) and inhibitors (boronic acid) or the cefoxitin-cloxacillin double disk synergy test (61). Apart from that, in this study we did not detect ESBL-producing strains nor the ESBL encoding gene *bla*_CTX−M_. ESBL is a group of enzymes with the ability to hydrolyze and cause resistance to oxyimino-cephalosporins and monobactams, but not to cephamycins or carbapenems, and that are inhibited by β-lactamase inhibitors (57). This group includes TEM, SHV, OXA, and CTX types (57). CTX-M ESBLs have increased its prevalence in the last decade in *E. coli* strains isolated from humans and animals (62, 63) and are the most common type of ESBL worldwide (64). Similarly, we did not detect any strain resistant to carbapenems nor harboring the *bla*_NDM−1_ gene. NDM-1 is capable to hydrolyze penicillins, cephalosporins, carbapenems, but not aztreonam, and its encoding gene is usually located in conjugative plasmids, representing a significant threat to public health worldwide (65). NDM-1 harboring *E. coli* strains have been isolated worldwide, including Chile, since its discovery in 2008 (66, 67). Nevertheless, to date there are no reports of its detection in *E. coli* strains isolated from animals in Chile.

Regarding polymyxins, we detected an 81.5% of colistin resistance. Colistin resistance was associated only with point mutations on chromosomal genes, until a plasmid-mediated colistin resistance gene, *mcr*-1, was identified in Chinese clinical and swine-isolated *E. coli* strains in late 2015 (68). Just 3 months after this finding, it was described that this gene was present in most continents and mainly in *E. coli* strains isolated from animals, environment, foodstuff, and infected and asymptomatic human carriers (69). To date, 10 different *mcr* genes have been reported, some of them even with variants (24, 70, 71). Food-producing animals have been highlighted as potential reservoirs of *mcr*-harboring strains, and together with the fact that colistin is currently being used as the last resort against carbapenem-resistant Gram-negative bacteria in humans, this phenomenon poses a major threat to public health. To date, in Chile there is only one report of a human clinical isolate of colistin-resistant *E. coli* harboring the *mcr*-1 gene (72), and no reports of strains isolated from animal reservoirs harboring this gene. In Chile, the use of colistin is approved only for therapeutic purposes in cattle, poultry, and swine (73). According to the MIC values determined here, it is evident that most of the strains were phenotypically resistant to colistin, but it was not possible to associate these high levels of colistin resistance (MIC_50_ > 4 μL/mL) with the presence of any of the *mcr* genes assessed. Similarly, Luo et al. detected a 47.5% of colistin resistant clinical isolates of *E. coli* in China that did not harbor any mobile *mcr* genes (74). This phenotypic resistance in absence of colistin-encoding mobile elements may be due to chromosomal mutations in the *mgr*B, *pho*PQ, and *pmr*AB genes, which would confer lipid A modifications (74).

Phenicol resistance is mainly due to the presence of chloramphenicol acetyltransferases encoded by *cat* genes that inactivate chloramphenicol but no other related compounds such as florfenicol; and to a lesser extent due to efflux pumps encoded by *cml* genes, among others (75). These genes can be detected in a wide variety of Gram-negative bacteria, including STEC, and are often associated with mobile elements such as plasmids, that can be transferred between bacteria of different species and genera (22, 75). Chloramphenicol resistance levels were low in the non-O157 STEC strains examined here (14.8%), but lower than results reported in México (47) and Argentina (22), where 60 and 80% of STEC strains isolated from cattle and swine were resistant to this drug, respectively. Contrary to the phenotypic resistance observed, we detected the *cat*1 gene in 61.1% of STEC strains, detection similar to that reported in Argentina where 40% of the STEC strains isolated from cattle and swine harbored this gene (22).

Resistance to quinolones is a major concern worldwide, as these antimicrobials are critically important for human and veterinary medicine (55, 56). Here, we registered only three isolates resistant to enrofloxacin, but sensitive to ciprofloxacin, similar to that reported in STEC strains isolated from cattle in South Africa (76), where 7.4% of the strains were resistant to enrofloxacin and 12.6% to ciprofloxacin. This different susceptibility to ciprofloxacin and enrofloxacin could be due to the presence of efflux pumps, as different members of this antimicrobial family show selective affinity for these (77). Conversely, mutations in topoisomerase genes would generate non-selective resistance to quinolones (58). Our findings suggest a restricted use of these drugs in livestock, probably due to national policies that do not encourage the use of quinolones as the first line of treatment, unless there is no other therapeutic alternative available. National policies also require that when quinolones are used as secondary treatment, their selection is based on the results of a susceptibility analysis (78).

Tetracyclines are broad-spectrum antibiotics that inhibit peptide elongation (75), and are considered of critical importance and high importance for veterinary and human medicine, respectively (55, 56). Tetracycline resistance occurs most frequently by the acquisition of genes that code for efflux pumps, ribosomal protection proteins, or by enzymatic inactivation. Many of the genes involved in these mechanisms are associated with mobile elements, and most of them encode resistance efflux proteins (75). Here, we detected tetracycline resistance in only a 3.7% of the isolated STEC strains, but *tet*A and *tet*B genes were detected in 100 and 94.4% of the strains, respectively. Tetracycline resistance levels reported here are lower than those reported previously in Ireland, where 82% of the non-O157 STEC strains isolated from cattle were resistant to tetracycline, while the *tet*A gene was detected in 60% of these strains (18). More recently, Colello et al. registered a 100% of tetracycline resistance in STEC strains isolated from cattle and swine, but the presence of *tet*A and *tet*B genes in a 20 and 40%, respectively (22). This contradiction between phenotypic resistance and low detection of *tet* genes could be explained by the existence of other 43 tetracycline resistance genes (75), that could be present in those strains.

Of the detected AMR genes, *aac(6)-Ib, bla*_TEM−1_, *bla*_*amp*__C_, and *cat*1 were located closed together in the correspondence map for resistance genes, suggesting that their presence was correlated among bovine isolates. In other words, when one of these four genes are present in an isolate, the others are likely to be present as well. The presence of the *aac(3)-IIa* gene seems to be less correlated to the presence of genes *aac(6)-Ib, bla*_TEM−1_, *bla*_AmpC_, and *cat*1. Additionally, the presence of genes *intI1* and *intI2* in all swine isolates explains their importance in the MCA, but interpretations must be made with caution due to the small number of swine samples in this study.

An interesting observation was the high detection levels for certain resistance genes with few or none STEC strains showing the associated phenotypic resistance, such as *tet*A (100%) and *tet*B (94.4%) vs. a 3.7% of resistance against doxycycline; *dfr*A1 (100%), *aac(3)-IIa* (11.1%), and *aac(6)-Ib* (88.9%) with no strains resistant to trimethoprim and aminoglycosides. One possible explanation to this is the lack of promoters or mutations in these regions, thus preventing gene expression (79). According to these authors, the accumulation and retention of deleterious mutations in resistance genes is higher in bacterial populations growing in absence of antimicrobial selection pressure than in bacterial populations under intense antimicrobial pressure (79). Nevertheless, other authors have shown that some of these inactivated resistance genes could be re-expressed due to genetic modifications or exposure to a selected drug, allowing the rapid reappearance of resistant phenotypes in previously antibiotic-susceptible strains (80). This fact highlights the need to detect AMR genes not only in phenotypically resistant isolates, but in all strains that could pose a risk to public health. Over time, random mutations should accumulate in gene sequences that encode resistance to rarely used drugs, because there would be fewer selection events resulting from the use of these antimicrobials (79). If so, we can hypothesize that the use of aminoglycosides, phenicols, tetracyclines, and trimethoprim used to be frequent in Chilean cattle and swine production. Nevertheless, current amount of sales of these compounds for therapeutic use in terrestrial productive animals in Chile is not one of the largest, being surpassed by macrolides, pleuromutilin, and penicillins (81). In Chile, the use of chloramphenicol as a growth promoter is prohibited since 1996 (82), and the use of any kind of antimicrobials for this purpose since 2006 (83). Probably, strains adapted to selective pressure by these antimicrobials became dominant in STEC populations, and now with the reduction in the use of these drugs, some resistance genes mutated and became pseudogenes. Moreover, AMR carries a fitness cost that can reduce bacterial growth rate, competitive ability, or virulence. This high cost could generate selection against resistance, being a relevant factor in the evolutionary dynamics of resistance, especially when bacteria encounter an antibiotic-free environment (84). Taken together, this evidence could explain the high detection levels of bacteria that harbor AMR genes without the associated resistant phenotype.

Regarding detection of class 1 and class 2 integrons, here we detected them in a 5.6% each, and only in strains isolated from swine. Integrons are natural mobile capture systems and assembly platforms that allow bacteria to incorporate gene cassettes and further convert them into functional proteins through proper expression, playing an essential role in the spread of a wide range of resistance genes among different bacterial populations (85). Kennedy et al. detected the presence of class 1 integrons in 21% of non-O157 STEC strains isolated from cattle, while no class 2 integrons were detected (18). More recently, class 1 integrons were detected in 0.8% of STEC strains analyzed (22). The high MDR observed here in the STEC strains isolated from swine could be due to combined presence of class 1 and class 2 integrons.

Some authors have also demonstrated an association between AMR and virulence in STEC strains. Thus, Mora et al. reported higher resistance levels in non-O157 STEC strains isolated from humans, cattle, sheep, and food in Spain that harbored the *eae* gene (86). This gene codes for intimin, it is involved in the attachment/effacing lesions of intestinal epithelia and it is often found in strains related to HUS (6). Later, Buvens et al. reported that non-O157 STEC strains isolated from humans, animals, food, and the environment in Belgium, which harbored the *eae* gene, presented higher resistance against streptomycin, kanamycin, and tetracycline than intimin negative non-O157 STEC strains (50). In this study, only one non-O157 strain harbored the *eae* gene (strain 7), but showed phenotypic and genotypic resistance like the other strains.

STEC strains do not only represent a major risk for public health due to the number of infections in humans and their sequels, but also due to the severe economic losses of the food industry due to the withdrawal of contaminated food products. Presence of STEC strains with phenotypic and/or genotypic resistance is especially relevant when it comes to establishing new antibiotic-based therapies for early-stage STEC infections in humans, which can help prevent serious sequelae (23). In addition, official control of STEC presence in food is progressing worldwide, through the introduction of a discussion paper and project document on “Control of Shiga toxin-producing *Escherichia coli* (STEC) in beef, unpasteurized milk and cheese produced from unpasteurized milk, leafy greens, and sprouts,” presented by Chile, the United States of America, and Uruguay at the 50th Session of the Codex Committee on Food Hygiene (87). This joint strategy suggests that, in the short term, not only the presence of this pathogen must be of mandatory surveillance, but also its AMR determinants.

Finally, our results show that non-O157 STEC strains present in the animal component of the animal-human interface in the Metropolitan region of Chile exhibit phenotypic and genotypic resistance against critical and important antimicrobials for human and veterinary use, representing a major threat for public health. Furthermore, these strains could have a competitive advantage in the presence of antimicrobial selective pressure, leading to an increase in food contamination. This study highlights the need for coordinated local and global actions concerning antimicrobial use in food animal production.

## Data Availability Statement

The datasets presented in this article are not readily available because they are part of a whole-genome sequencing study, not yet published. Requests to access the datasets should be directed to Dr. Nicolás Galarce (ngalarce@ug.uchile.cl).

## Ethics Statement

The animal study was reviewed and approved by the Comité Institucional de Cuidado y Uso de Animales of the Universidad de Chile (permit code 17083-VET-UCH) for obtaining rectal samples from cats and dogs. Ethical approval for samples of pigs and cattle from abattoirs was not required according to national/local legislation.

## Author Contributions

NG and CB contributed to the conception and design of the study. NG, CB, LL, NL, EP-O, GG-R and HB-T contributed with resources to the study. FS, BE, VF, RR, DF-C, and AV-L performed the laboratory analyses. RA-M and GA performed the statistical analysis. NG wrote the first draft of the manuscript. RA-M and GA wrote sections of the manuscript. All authors contributed to the article and approved the submitted version.

## Conflict of Interest

The authors declare that the research was conducted in the absence of any commercial or financial relationships that could be construed as a potential conflict of interest.
